# Characterization of the pathogenesis and immune response to *Listeria monocytogenes* strains isolated from a sustained national outbreak

**DOI:** 10.1038/s41598-019-56028-3

**Published:** 2019-12-20

**Authors:** Pallab Ghosh, Yan Zhou, Quentin Richardson, Darren E. Higgins

**Affiliations:** Department of Microbiology, Blavatnik Institute, Harvard Medical School, Boston, MA USA

**Keywords:** Pathogens, Bacterial infection

## Abstract

*Listeria monocytogenes* is an intracellular pathogen responsible for listeriosis, a foodborne disease that can lead to life-threatening meningitis. The 2011 *L. monocytogenes* cantaloupe outbreak was among the deadliest foodborne outbreaks in the United States. We conducted *in vitro* and *in vivo* infection analyses to determine whether strains LS741 and LS743, two clinical isolates from the cantaloupe outbreak, differ significantly from the common laboratory strain 10403S. We showed that LS741 and LS743 exhibited increased virulence, characterized by higher colonization of the brain and other organs in mice. Assessment of cellular immune responses to known CD8^+^ T cell antigens was comparable between all strains. However, pre-existing immunity to 10403S did not confer protection in the brain against challenge with LS741. These studies provide insights into the pathogenesis of clinical isolates linked to the 2011 cantaloupe outbreak and also indicate that currently utilized laboratory strains are imperfect models for studying *L. monocytogenes* pathogenesis.

## Introduction

*Listeria monocytogenes* is a Gram-positive bacterium commonly found in the soil. *L. monocytogenes* is also a foodborne intracellular pathogen whereby eating contaminated food can lead to listeriosis, a severe invasive disease that most often occurs in pregnant women, newborns, the elderly, and immunocompromised individuals^1,2^. No vaccine currently exists for *L. monocytogenes* and the ability of bacteria to effectively spread to distal organs such as the brain or the placenta may lead to life-threatening meningitis or septic abortion^3,4^. *L. monocytogenes* is among the leading causes of death by a foodborne pathogen in the U.S., with case fatality rates from listeriosis as high as 20‒30%, the highest for all foodborne pathogens^5^.

Generally, foods such as soft cheeses, deli meats and processed seafood contaminated with *L. monocytogenes* have been involved in various listeriosis outbreaks, however, an increasing number of recent outbreaks have involved fresh produce as the source for infection^6,7^. The 2011 *L. monocytogenes* cantaloupe outbreak in the U.S. was among the deadliest foodborne outbreaks in the last several decades^8^. A total of 147 people were sickened and 33 people died from *L. monocytogenes* contaminated cantaloupes. This was also the largest U.S. listeriosis outbreak reported to date involving *L. monocytogenes* serotype 1/2a and 1/2b strains, both identified from contaminated cantaloupes^9,10^. Multilocus sequence typing, a DNA sequence-based discriminatory typing method used for the evaluation of intraspecies genetic relatedness, identified the serotype 1/2a strains (lineage II) as clonal complex 7 that also includes standard laboratory reference strain *L. monocytogenes* 10403S, whereas the serotype 1/2b strains (lineage I) belonged to clonal complex 5^11–13^.

Recently, a large-scale European study assessed *L. monocytogenes* strains of clinical and food origin and reported that *L. monocytogenes* clones associated with severe human infections are hypervirulent in mice compared to the *L. monocytogenes* strains isolated from food sources^14^. However, bacterial infection phenotypes associated with the *L. monocytogenes* strains isolated from U.S. listeriosis outbreaks remain poorly understood and warrant more detailed investigation. Furthermore, *L. monocytogenes* has also been used for over five decades as a model organism to study the cellular immune response to intracellular pathogens^15^. Infection of mice with *L. monocytogenes* induces strong CD8^+^ T cell responses, which play a primary role in providing protection against secondary infection^16,17^. The study of the immune response to *L. monocytogenes* in the mouse infection model continues to provide numerous insights to understanding the role of cytokines, receptors, and adaptor molecules that influence the generation of CD8^+^ T cell responses during infection^18–20^.

In this study, we focused on characterizing virulence and the host immune response to clinical isolates of *L. monocytogenes* strains associated with the 2011 listeriosis outbreak involving contaminated cantaloupes. Our data demonstrated that the *L. monocytogenes* outbreak strains are highly invasive in human cells and more virulent in mice compared to the *L. monocytogenes* 10403S laboratory strain. We showed that *L. monocytogenes* outbreak strains possess an enhanced ability to invade the brain. Moreover, we compared CD8^+^ T cell responses elicited in BALB/c mice by a representative clinical isolate and the 10403S strain. The CD8^+^ T cell responses to known bacterial antigens were comparable between 10403S and the representative clinical isolate. However, immunization with 10403S did not confer complete protection against challenge with the clinical isolate, presumably due to the enhanced virulence of the clinical isolate strain. These studies demonstrate that while there are numerous similarities amongst *L. monocytogenes* strains, the currently utilized laboratory strains do not provide an ideal model to fully understand the mechanisms of *L. monocytogenes* pathogenesis.

## Results

### Cantaloupe outbreak-associated *L. monocytogenes* strains are more efficient in host cell invasion and cell-to-cell spread

**Figure 1:**
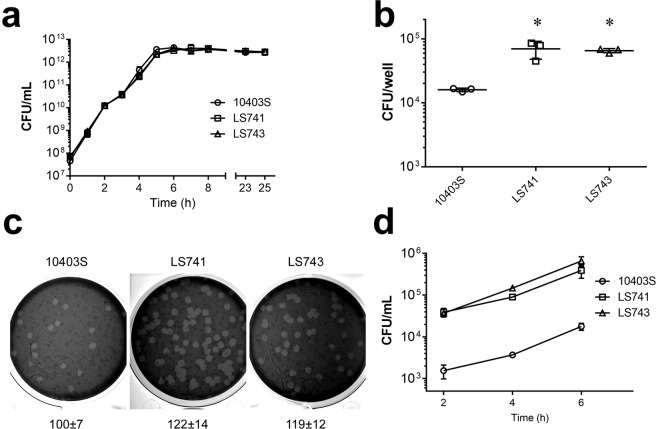
*In vitro* characterization of *L. monocytogenes* strains. (**a**) Growth of *L. monocytogenes* strains in BHI broth. The indicated strains were grown at 37 °C for 25 hours in BHI broth and the number of bacteria was determined at the indicated times. Data represent the mean ± SD colony forming units (CFU) per milliliter for three experiments performed in duplicate with similar results. (**b–d**) Intracellular infections in HeLa cells. (**b**) HeLa cells were infected with the indicated *L. monocytogenes* strains for 1 hour prior to intracellular bacteria being quantified by gentamicin protection assay at 2 hours post-infection. Data represent the mean ± SD CFU per well for one of three experiments performed in triplicate with similar results. *P < 0.05. (**c**) Plaque formation by *L. monocytogenes* strains. HeLa cells were infected with the indicated strains. After 1 hour, the infected monolayers were washed and overlaid with medium containing gentamicin and 0.7% agarose. At 72-hours post-infection, monolayers were stained with neutral red to allow for visualization of plaques. Wells were photographed and plaque sizes were measured at 96 hours post-infection. Numbers indicate the mean percentage plaque sizes ± SD normalized to 10403S from three independent experiments measuring >10 plaques/experiment. (**d**) Intracellular growth in HeLa cells. HeLa cells were infected with the indicated strains for 1 hour. Gentamicin was then added and at 2-hour time intervals post-infection, HeLa cells were lysed and intracellular bacteria were enumerated by plating dilutions of lysates. Data presented is the mean ± SD of three independent experiments performed in duplicate.

We initially characterized 7 clinically isolated *L. monocytogenes* strains associated with the U.S. 2011 cantaloupe outbreak. These strains consisted of five serotype 1/2a and two serotype 1/2b strains isolated from infected patients^9,10^ (Supplementary Table S1). We first determined if there were any differences in extracellular growth rates between the clinically isolated strains and the commonly used 10403S laboratory strain^21–23^ when bacteria were grown in BHI medium. There was no major difference in the growth rate among all of the strains examined over an 8-hour growth period (Supplementary Fig. S1). We next selected two representative *L. monocytogenes* strains (LS741 and LS743) to further characterize and compare to 10403S. *L. monocytogenes* LS741 was selected as it was one of the two available serotype 1/2b strains (Supplementary Table S1), and *L. monocytogenes* LS743 was selected among the serotype 1/2a strains since a draft genome sequence is available^24^. We next confirmed that similar growth rates between *L. monocytogenes* strains 10403S, LS741 and LS743 were observed when bacteria were grown in BHI broth over 25 hours (Fig. 1a). To examine the capacity for host cell invasion by the outbreak strains, we performed gentamicin protection assays using human HeLa epithelial cells^25,26^. As shown in Fig. 1b, infection of HeLa cells by *L. monocytogenes* strains LS741 and LS743 resulted in a >5-fold increase in intracellular bacteria compared to infection by *L. monocytogenes* 10403S. Multiple studies have reported that flagellar motility plays an important role for the ability of *L. monocytogenes* to invade host epithelial cells^27,28^. We therefore compared flagellar motility of the outbreak strains to 10403S and observed no major differences in flagellar motility (Supplementary Fig. S2), suggesting that the increased invasion of HeLa cells observed with the outbreak strains (Fig. 1b) was not attributed to an increase in flagellar motility. We then determined the efficiency of plaque formation by the *L. monocytogenes* outbreak strains. The LS741 and LS743 strains produced an increased number of plaques that averaged ~120% of the diameter of those produced by *L. monocytogenes* 10403S (Fig. 1c), suggesting not only an increase in invasive capacity, but also an increase in the ability to spread cell-to-cell within host cells. Next, to examine the ability of the outbreak strains to replicate inside of host cells, we performed intracellular infection assays in HeLa cells (Fig. 1d). As also shown in Fig. 1b, we observed increased invasion by the LS741 and LS743 strains compared to 10403S at the initial 2-hour post-infection time point.

However, we did not observe any differences in the intracellular replication rate between both outbreak strains and 10403S (Fig. 1d). Taken together, these data indicated an increased ability of the *L. monocytogenes* outbreak strains LS741 and LS743 to invade host cells and spread cell-to-cell compared to 10403S, yet the intracellular replication rate was similar among all strains.

### *L. monocytogenes* cantaloupe outbreak-associated strains display increased virulence in mice

**Figure 2:**
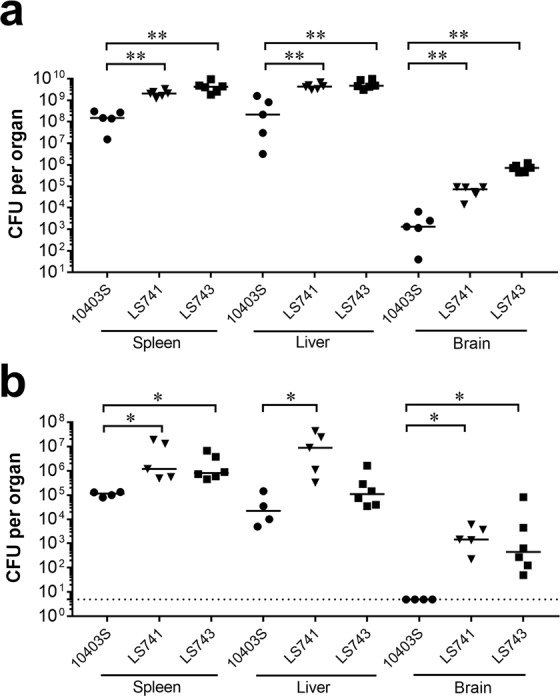
*L. monocytogenes* outbreak strains exhibit increased virulence in mice. BALB/c mice were infected intravenously with 1–2 × 10^4^ CFU (**a**) or orogastrically inoculated with 3–4 × 10^9^ CFU of *L. monocytogenes* 10403S, LS741 or LS743 (**b**). At 72 hours post-infection the liver, spleen, and brain of each mouse were collected and the bacterial burden determined. Horizontal lines indicate median values. The Mann-Whitney test was used to assess statistical significance between bracketed groups: *P < 0.05, **P < 0.01. The dotted line in panel b indicates a limit of detection of 5 CFU per organ.

To assess *in vivo* virulence, we evaluated infection by *L. monocytogenes* using a murine infection model. We first infected BALB/c mice intravenously with 1–2 × 10^4^ CFU of *L. monocytogenes* strains 10403S, LS741, or LS743 and the number of bacteria present in the spleen, liver and brain of each mouse was determined 72 hours post-infection. Mice infected with LS741 and LS743 showed an over 1-log increase in colonization of the spleen and liver compared to the 10403S bacterial burden (Fig. 2a). Furthermore, compared to infection with 10403S, mice infected with strains LS741 and LS743 showed, respectively, a 1.6-log and a 2.6-log increase in colonization of the brain (Fig. 2a). PrfA is a transcriptional activator of numerous virulence genes in *L. monocytogenes*. Specific point mutations in PrfA (PrfA* mutants) can result in constitutive activation of PrfA-regulated virulence genes. Prior studies have shown increased virulence of PrfA* strains during infection of mice^21,29^. Since both LS741 and LS743 displayed increased virulence compared to 10403S during infection of mice, we determined the sequence of *prfA* in 10403S, LS741 and LS743. Amino acid sequence analyses (Supplementary Fig. S3) showed no difference in the PrfA sequences between the three *L. monocytogenes* strains and the absence of any known PrfA* mutations, thus precluding the possibility of a mutation in PrfA being associated with the increased virulence of these outbreak strains.

We next determined the virulence of the *L. monocytogenes* strains by infecting BALB/c mice orogastrically with 3–4 × 10^9^ CFU bacteria. Similar to intravenous infection, infection with *L. monocytogenes* 10403S resulted in lower bacterial counts in the liver and spleen at 72 hours post-infection (Fig. 2b). Interestingly, bacterial counts in the brain were not detectable following infection with 10403S (limit of detection = 5 CFU) whereas significantly higher bacterial colonization was observed for LS741 (3.2-logs) and LS743 (2.8-logs) in the brain (Fig. 2b). Taken together, these results indicated that the *L. monocytogenes* LS741 and LS743 outbreak strains are more pathogenic than 10403S and in particular, invade the brain to a greater extent during infection in mice.

The difference in pathogenic potential between 10403S and a representative outbreak strain (LS741) was further assessed by determining the lethal dose (LD_50_) values following intravenous infection of BALB/c mice^30^. The LD_50_ value of *L. monocytogenes* strain 10403S was determined to be 1 × 10^4^ CFU, consistent with prior reports^31^. The LD_50_ of LS741 was determined to be ~3 × 10^3^ CFU. Therefore, consistent with an increase in bacterial burdens in organs (Fig. 2), LS741 demonstrated an overall increase in virulence capacity as determined by LD_50_ values.

### Listeriolysin O is a principal virulence determinant in cantaloupe outbreak-associated strain LS741

**Figure 3:**
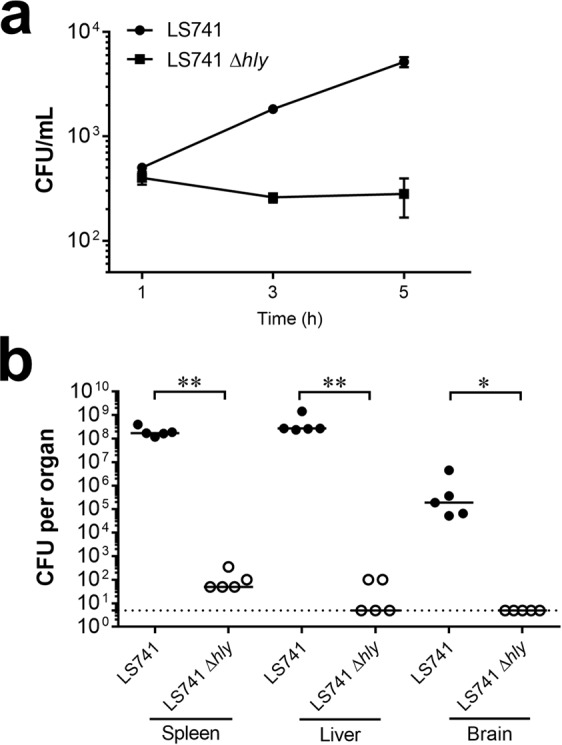
Listeriolysin O is required for *L*. *monocytogenes* strain LS741 intracellular infection and virulence. (**a**) Intracellular growth in HeLa cells. HeLa cells were infected with the indicated strains for 1 hour. Gentamicin was then added and at 2-hour intervals post-infection, HeLa cells were lysed and intracellular bacteria were enumerated by plating dilutions of lysates. Data presented is the mean ± SD of three independent experiments performed in duplicate. (**b**) *L*. *monocytogenes* LS741 Δ*hly* is significantly attenuated in mice. BALB/c mice were infected intravenously with *L. monocytogenes* LS741 or LS741 Δ*hly* bacteria (1–2 × 10^4^ bacteria/animal). At 72 hours post-infection the liver, spleen, and brain of each mouse were collected and the bacterial burden determined. Horizontal lines indicate median values. The Mann-Whitney test was used to assess statistical significance between bracketed groups: *P < 0.05, **P < 0.01. The dotted line indicates a limit of detection of 5 CFU per organ.

In the 2011 cantaloupe outbreak, both serotype 1/2a and 1/2b *L. monocytogenes* strains were shown to cause human listeriosis^9^. Importantly, *L. monocytogenes* serotype 1/2b strains contributed to 27% of the listeriosis cases that resulted in 30% fatalities in this outbreak^8^. In an effort to understand the virulence attributes of the outbreak-associated strains, we determined whether standard molecular biology techniques would be amenable to genetic manipulation of a representative outbreak strain (LS741). We also examined the contribution of the principal virulence determinant listeriolysin O (LLO) for intracellular infection by LS741. LLO, the gene product of the *hly* gene, is a secreted pore-forming cytolysin that mediates bacterial escape from the membrane-bound vacuole formed following *L. monocytogenes* invasion of a host cell^32^. We initially compared hemolytic activity present in culture supernatants as a measure of LLO activity^32^. Culture supernatants of the LS741 strain exhibited hemolytic activity equivalent to that of 10403S (32.3 ± 3.1 and 40.4 ± 5.2 hemolytic units, respectively). Furthermore, as shown in Fig. 3a, the LLO-negative strain (LS741 Δ*hly*) was unable to escape from primary vacuoles and grow within the host cell cytosol of HeLa cells during the 5-hour infection period examined. A similar requirement of LLO for intracellular replication in HeLa cells was also observed during infection by 10403S Δ*hly* bacteria (Supplementary Fig. S4)^26^. We next investigated whether LLO was important for *L. monocytogenes* LS741 virulence. Consistent with earlier reports, our data showed that deletion of the *hly* gene resulted in attenuation of virulence during infection of BALB/c mice^33,34^. The LS741 Δ*hly* strain was attenuated in colonization in the spleen and liver, displaying a 6–7 log reduction of bacteria compared to the LS741 parental strain (Fig. 3b), whereas no LS741 Δ*hly* bacteria were detected (limit of detection = 5 CFU) in the brains of infected animals (Fig. 3b). Overall, these data confirmed that LLO plays an important role in the pathogenicity of the outbreak-associated *L. monocytogenes* strain LS741 and that existing molecular biology techniques for genome manipulation are amenable in this outbreak strain.

### Infection of mice with 10403S or outbreak strain LS741 induce similar levels of *Listeria*-, LLO- and p60-specific IFN-γ producing CD8^+^ T cells

**Figure 4:**
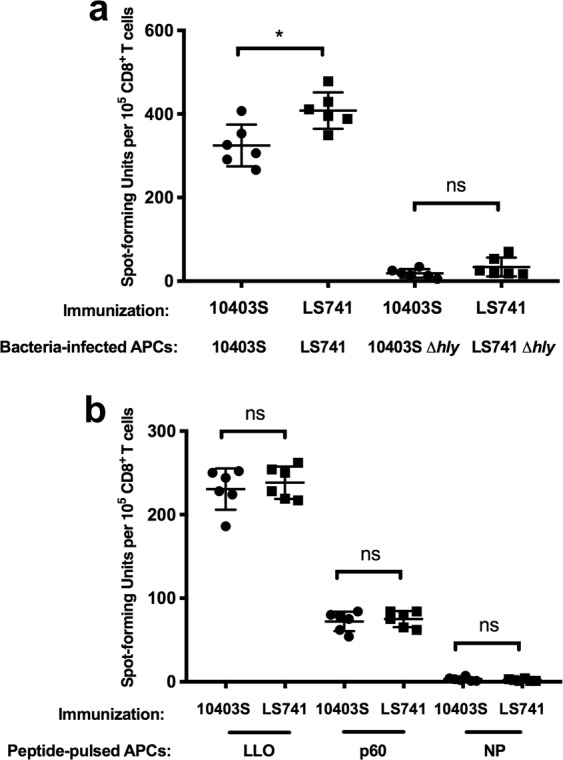
Characterization of CD8^+^ T cell responses in LS741 or 10403S immunized mice. BALB/c mice were immunized intravenously with an ~0.1 LD_50_ dose of 10403S (1 × 10^3^ CFU) or LS741 (300 CFU). Seven days post-immunization, mice were euthanized and spleens were collected for *L*. *monocytogenes*-specific CD8^+^ T cell isolation. BALB/c mouse bone marrow-derived macrophages were seeded as APCs on IFN-γ antibody coated plates (1 × 10^5^ APCs/well). APCs were pulsed with 5 × 10^5^ CFU of the indicated *L. monocytogenes* strain (**a**) or a 100 nM concentration of LLO, p60 or influenza NP peptides (**b**). Following a 4-hour incubation (**a**) or an incubation of 1–2 hours (**b**), 1 × 10^5^ CD8^+^ T cells from mice immunized with the indicated strain were added for analysis by IFN-γ ELISPOT. Data shown represents the mean ± SD of three replicates and are combined from two separate experiments. The Student’s *t*-test was used to assess statistical significance between bracketed groups: *P < 0.05, ns = not statistically significant.

The murine model of *L. monocytogenes* infection has been used for over five decades to study aspects of acquired cell-mediated immunity to intracellular infection^18^. A critical component of protective immunity to *L. monocytogenes* is the generation of cytotoxic CD8^+^ T cells^16,35^. The primary functions of these *Listeria*-specific CD8^+^ T cells are lysis of infected host cells and secretion of IFN-γ for activation of host macrophages^36,37^. BALB/c mice infected with *L. monocytogenes* have been shown to develop protective CD8^**+**^ T cell responses against peptides encompassing amino acids 91–99 of LLO and amino acids 217–225 of the p60 protein^38–41^. To compare the ability of *L. monocytogenes* strains to elicit pathogen-specific CD8^+^ T cells during *in vivo* infection, BALB/c mice were infected with a subclinical (0.1 LD_50_) dose of 10403S or LS741. Seven days post-immunization, the quantity of *L. monocytogenes*-specific CD8^+^ T cells was assessed. This assessment was performed using an enzyme-linked immunospot (ELISPOT) assay to determine the frequency of cytokine (IFN-γ) secreting CD8^+^ T cells that were specific to *L. monocytogenes* or peptides derived from *L. monocytogenes*-specific antigens. As shown in Fig. 4a, immunization of mice with LS741 resulted in the priming of an increased number of *L. monocytogenes*-specific CD8^+^ T cells compared to immunization with 10403S (408 spot-forming units (SFU) compared to 325 SFU, respectively). Furthermore, consistent with prior studies showing a requirement of vacuole escape and cytosolic access to allow for conventional MHC class I processing^36,42^, infection of antigen presenting cells (APCs) with LLO-negative *L. monocytogenes* strains did not result in activation of IFN-γ producing *L. monocytogenes*-specific CD8^+^ T cells (Fig. 4a).

To further determine the frequency of antigen-specific CD8^+^ T cells primed during immunization of mice, APCs pulsed with peptides that were either LLO-, p60-, or influenza virus nucleoprotein (NP)-specific were used in the ELISPOT assay. As shown in Fig. 4b, both LLO- and p60-specific IFN-γ producing CD8^+^ T cells were primed at similar frequencies following immunization with either 10403S or LS741. Consistent with prior reports, our results showed that LLO-specific CD8^+^ T cells were primed at a higher frequency than p60-specific CD8^+^ T cells^43,44^ (Fig. 4b). As expected, IFN-γ producing CD8^+^ T cell responses against the non-specific control influenza NP peptide were not primed following immunization with *L. monocytogenes*. Collectively, these data indicated that immunization with an equivalent infectious dose (0.1 LD_50_) of LS741 resulted in the generation of a greater number *L. monocytogenes-*specific CD8^+^ T cells than immunization with 10403S (Fig. 4a). However, the frequency of LLO- and p60-specific CD8^+^ T cells primed following immunization were similar between the two strains (Fig. 4b).

### Pre-existing immunity to *L. monocytogenes* 10403S does not provide complete protection to challenge with outbreak strain LS741

*In vitro* and *in vivo* infection studies indicated that *L. monocytogenes* LS741 was more virulent than the well-characterized 10403S strain (Figs. 1, 2 and LD_50_ determination). Nonetheless, immunization of mice with an equivalent infectious dose of either strain stimulated similar levels of *L. monocytogenes* antigen-specific CD8^+^ T cells (Fig. 4b). Given the ubiquitous nature of *L. monocytogenes*, it is probable that some individuals sickened in the 2011 cantaloupe outbreak may have been previously exposed to *L. monocytogenes*. To examine whether prior immunization with a less virulent *L. monocytogenes* strain provides protection to a secondary challenge with a *L. monocytogenes* outbreak strain (cross-strain protection), we immunized mice with a subclinical (0.1 LD_50_) dose of either 10403S or LS741 and subsequently challenged animals 28 days later with an equivalent lethal dose (1 × 10^5^ CFU) of the same or reciprocal *L. monocytogenes* strain. We then assessed immune protection by measuring the reduction in bacterial burdens in organs of mice 48 hours post-challenge.

**Figure 5:**
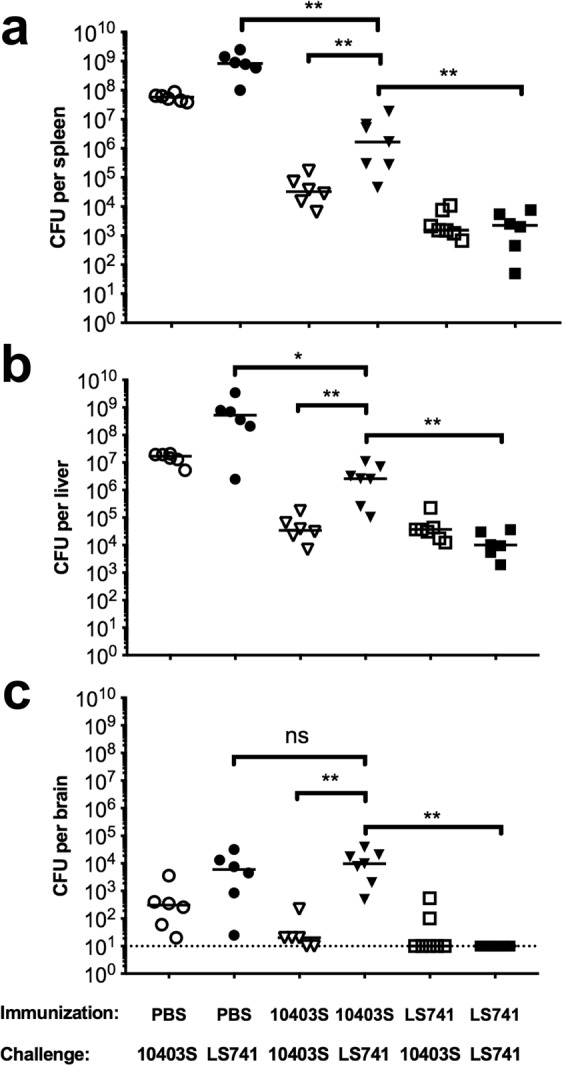
Pre-existing immunity to *L. monocytogenes* 10403S provides limited protection to challenge with outbreak strain LS741. BALB/c mice were immunized with approximately 0.1 LD_50_ of the indicated strains (1 × 10^3^ CFU of 10403S or 300 CFU of LS741) or PBS. Twenty-eight days later, immunized mice were challenged with 1 × 10^5^ CFU of the indicated strains. At 48 hours post-challenge, mice were euthanized and the spleen (**a**), liver (**b**) and brain (**c**) of each mouse was collected and the bacterial burden determined. Horizontal lines indicate median values. The Mann–Whitney test was used to assess statistical significance between bracketed groups: *P < 0.05, **P < 0.01, ns = not statistically significant (*P* = 0.45). The dotted line in panel c indicates a limit of detection of 10 CFU per brain.

As shown in Fig. 5, mice mock-immunized with PBS and then challenged with 1 × 10^5^ CFU of 10403S or LS741 displayed similar bacterial burdens in organs at 48 hours post-challenge as did mice that were infected with 1–2 × 10^4^ CFU displayed at 72 hours post-infection (Fig. 2a). These results again demonstrated a higher bacterial burden in all organs when PBS immunized mice were infected with LS741 compared to 10403S (Fig. 5; PBS immunized). Mice immunized with 10403S and challenged with 10403S exhibited significant immunity compared to PBS immunized/10403S-challenged mice, resulting in a >3-log reduction in bacterial burden in the spleen, a >2-log reduction in bacterial burden in the liver, and a >1-log reduction in bacterial burden in the brain to just above the limit of detection (Fig. 5). Mice immunized with 10403S and then challenged with LS741 also exhibited significant immunity compared to PBS immunized/LS741-challenged mice, resulting in an ~3-log reduction in bacterial burden in the spleen and an ~2-log reduction in bacterial burden in the liver (Fig. 5a,b). Interestingly, mice immunized with 10403S and then challenged with LS741 exhibited no reduction in bacterial burden in the brain compared to mice immunized with PBS and challenged with LS741 (Fig. 5c). Thus indicating no protective immunity to challenge with LS741 in the brain despite the same animals demonstrating measurable protective immunity in other organs (Fig. 5a,b). Furthermore, mice immunized with LS741 and challenged with 10403S exhibited significant immunity compared to PBS immunized/10403S-challenged mice, resulting in a >4-log reduction in bacterial burden in the spleen, an ~3-log reduction in bacterial burden in the liver, and a median reduction in bacterial burden in the brain down to the limit of detection (Fig. 5a–c). Similarly, mice immunized with LS741 and then challenged with LS741 displayed significant protection compared to PBS immunized/LS741-challenged mice, resulting in a >5-log reduction in bacterial burden in the spleen and a >4-log reduction in bacterial burden in the liver (Fig. 5a,b). Moreover, mice immunized with LS741 and then challenged with LS741 exhibited complete protection to colonization of the brain with no bacteria detected in the brains of any of the animals analyzed. This result indicated an ~4-log reduction in bacterial burden in the brain compared to mice immunized with PBS or 10403S and then challenged with LS741 (Fig. 5c).

Collectively, these results indicated that as determined by reduction in bacterial burdens in the spleen and liver, prior infection with the laboratory-adapted 10403S strain provided significant protection to a secondary infection with a lethal dose of the LS741 outbreak strain. However, prior immunization with 10403S provided no protection for colonization of the brain in LS741-challenged animals. Immunization with LS741 resulted in a similar or increased reduction of bacterial burdens in all organs following challenge with 10403S as did immunization and challenge with 10403S. Notably, immunization with LS741 compared to 10403S-immunization, resulted in an additional 2-log increase in the reduction of bacterial burdens in the spleen and liver and complete elimination of bacterial colonization of the brain following challenge with LS741.

## Discussion

*L. monocytogenes* strains are heterogenous and display a range of virulence phenotypes that are most likely due to intraspecies virulence-related differences between strains^1,45^. Recently, a study from Europe on epidemic *L. monocytogenes* isolates revealed that the serotype 4b encoded *Listeria* pathogenicity island 4 (LIPI-4) could be partly responsible for *L. monocytogenes* infection of the brain and placenta in mice^14^. However, the absence of LIPI-4 from other infection-associated *L. monocytogenes* hypervirulent strains suggested that selective bacterial factors are responsible for contributing to tissue tropism^14^. Through specific genetic and genome-wide analyses, prior studies demonstrated that *L. monocytogenes* isolates from the 2011 U.S. cantaloupe outbreak belong to two clonal groups: serotype 1/2a (lineage II) and 1/2b (lineage I)^9,12^. Since the characterization of these outbreak strains remains poorly studied, we investigated the pathogenicity and immune response to representative clinical isolates LS741 and LS743 compared to the commonly used *L. monocytogenes* laboratory strain 10403S (Supplementary Table S1).

We show that *in vitro* growth in broth culture for all of the outbreak strains and 10403S is similar (Fig. 1a and Supplementary Fig. S1). Additional *in vitro* infection experiments indicated that the outbreak-associated strains have an enhanced ability to invade human cells and spread intracellularly (Fig. 1b,c). Furthermore, the increase in invasion efficiencies of LS741 and LS743 were not attributed to an increase in flagellar motility compared to 10403S (Supplementary Fig. S2)^27^. However, at this time, we cannot rule out the possibility that differential expression of previously identified *L. monocytogenes* invasion determinants contributes to the increased invasive capacity of LS741 and LS743 compared to 10403S. Despite the increase in invasion, the intracellular replication rate of the outbreak strains was similar to that of 10403S (Fig. 1d). To understand the virulence differences between the outbreak-associated isolates and 10403S, we conducted *in vivo* infection experiments in mice. Infection of mice intravenously or orogastrically with LS741 or LS743 resulted in higher bacterial colonization in all mouse organs examined compared to infection with 10403S (Fig. 2). These results suggest that the increased invasion and cell-to-cell spread capability of LS741 and LS743 (Fig. 1b,c) mediates an enhanced ability to colonize multiple organs during infection of the host (Fig. 2). Most notable was the level of colonization of the brain following the natural oral infection route (Fig. 2b), where the outbreak strains demonstrated a 2–3-log increase in bacterial burden compared to undetectable levels of 10403S in the brain following oral infection. These data suggest that the outbreak-associated strains are more efficient in mechanisms mediating crossing of the blood-brain barrier to infect the brain (e.g. host cell invasion, cell-to-cell spread)^46^. Alternatively, an increase in the ability to survive and replicate within the infected host may lead to an overall increase in bacterial numbers that facilitates increased colonization of the brain in the absence of any specific enhanced mechanism of traversing the blood-brain barrier. Nonetheless, the increased bacterial burden observed in all organs examined independent of the infection route (Fig. 2) is consistent with the 3.3-fold decrease in the LD_50_ of outbreak strain LS741 compared to 10403S (~3 × 10^3^ CFU and 1 × 10^4^ CFU, respectively).

While the specific gene determinants that mediate the increase in virulence of LS741 and LS743 compared to 10403S are currently unknown, our respective sequence and hemolytic activity analyses of the principal virulence factors PrfA and LLO indicated no differences in protein sequence of PrfA (Supplementary Fig. S3) or increase in PrfA activity as measured by LLO hemolytic units. However, LLO was shown to be a conserved virulence factor as LS741 Δ*hly* bacteria were unable to escape the vacuole subsequent to host cell invasion to replicate in host cells (Fig. 3a). Furthermore, LS741 Δ*hly* bacteria were severely attenuated during *in vivo* infection with reduced or undetectable bacterial numbers in the organs of mice (Fig. 3b). Our studies do not preclude that expression differences or mutations in other identified or previously unknown virulence determinants play a role in the increased survival, invasion or cell-to-cell spread of the outbreak-associated strains. Because of our determination that outbreak strain LS741 is genetically tractable (Fig. 3), ongoing genetic studies are aimed at identifying the LS741 determinants that mediate increased invasion and cell-to-cell spread. Unfortunately, the complete genome sequences are not available for the *L. monocytogenes* outbreak strains LS741 and LS743. However, comparative analyses between the genome sequences of *L. monocytogenes* 10403S and serotype 1/2a and 1/2b clinical isolates from the 2011 U.S. cantaloupe outbreak for which complete genome sequences are available^10^, suggests there may be the presence of unknown genetic regions that are unique to these clinical isolates (e.g. LS741 and LS743). Although it is tempting to speculate that such genetic regions could play important roles in the pathogenesis of the outbreak strains, thorough experiments are needed to fully understand the basis of the increased virulence of the *L. monocytogenes* clinical isolates.

*L. monocytogenes* has been used for over five decades as a model organism to understand the development of acquired cellular immunity to intracellular pathogens^18^. These studies have shown that sterilizing immunity to infection requires the generation of CD8^+^ T cells^16^. Comparison of strain-specific T cell responses indicated a slight, but significant increase in the frequency of *Listeria*-specific CD8^+^ T cells primed following infection with LS741 as compared to 10403S (Fig. 4a). This increase in total *Listeria*-specific CD8^+^ T cells is consistent with an increase in bacterial organ burden (Fig. 2) leading to a higher frequency of antigen presentation and T cell priming. It is also possible that different antigen-specific CD8^+^ T cells are being primed at a higher frequency following infection with LS741 than infection with 10403S. However, since no significant difference was observed in priming of LLO- or p60-specific CD8^+^ T cells following LS741 or 10403S infection (Fig. 4b), this suggests that other strain-specific CD8^+^ T cells might be primed against antigens expressed by LS741 that are not expressed by 10403S. This hypothesis may also be supported by the cross-strain protection studies (Fig. 5). These data indicated that in our mouse model of infection, pre-existing immunity to LS741 provides significant or equivalent reduction in bacterial burdens in organs following challenge with a lethal dose of 10403S or LS741 (Fig. 5). Furthermore, pre-existing immunity to LS741 provided an equivalent (liver, *P* = 0.9714 and brain, *P *= 0.4779; Fig. 5b,c) or greater (spleen, *P* = 0.0047; Fig. 5a) reduction in bacterial burden following challenge with 10403S compared to mice immunized with 10403S. This result is consistent with data in Fig. 4a indicating that immunization with LS741 drives the generation of a higher frequency of *Listeria*-specific CD8^+^ T cells in the spleen.

In contrast, pre-existing immunity to 10403S did not provide equivalent reduction in bacterial burdens following challenge with LS741 as observed following challenge with 10403S (Fig. 5). The bacterial burden in organs of 10403S-immune mice was on average ~2-logs greater following challenge with LS741 than when challenged with 10403S (Fig. 5). Most striking was the colonization of the brain in LS741-challenged animals, where pre-existing immunity to 10403S provided no measureable reduction in bacterial burden following LS741 challenge. Bacterial burdens were similar as to mock-immunized (PBS-treated) animals that had been challenged with LS741 (Fig. 5c). Collectively, these data (Figs. 4 and 5) suggest that immune responses to shared CD8^+^ T cell antigens common to both 10403S and LS741 (e.g. LLO and p60) can provide measurable cross-strain protection in infected animals, but differences in the antigen repertoire and/or the increased invasive and dissemination capabilities of LS741 precludes measureable protection in the brain of 10403S-immune mice challenged with outbreak strain LS741. If extrapolated to infections in humans, our results suggest that increased virulence in conjunction with a potential lack of protective immunity in the event of prior exposure to *L. monocytogenes* contributed to the ability of LS741 and the other cantaloupe-associated strains to cause significant morbidity and mortality during the 2011 outbreak^8^.

To our knowledge, this is the first report to characterize the pathogenesis and immune response of *L. monocytogenes* clinical isolates from the sustained 2011 U.S. cantaloupe outbreak. Furthermore, our data suggests that with respect to studies using *L. monocytogenes* as a model to understand the mechanisms of cell-mediated immunity, both 10403S and LS741 provide tractable strains to gain insights given their similarities in extracellular growth, intracellular infection and priming of known *Listeria*-specific CD8^+^ T cells. However, our studies highlight that widely used *L. monocytogenes* laboratory strains may serve as incomplete models to study disease phenotypes relevant to *L. monocytogenes* infection in humans. Further studies into the pathogenesis of the genetically tractable LS741 clinical isolate strain may allow a better understanding of the pathogenic mechanisms of *L. monocytogenes* relevant to the increased virulence of human outbreak strains and may aid in the design of improved strategies for prevention of mortality due to listeriosis.

## Methods

### Bacterial strains

All bacterial strains used in this study are listed in Supplementary Tables S1 and S2 in the Supplementary Information. *L. monocytogenes* strains were grown in Brain Heart Infusion (BHI) medium (Difco). *E. coli* strains were grown in Lysogeny Broth (Luria–Bertani, LB) medium. Antibiotics (Sigma-Aldrich) were used at the following concentrations: streptomycin 100 μg/mL, nalidixic acid 50 μg/mL, carbenicillin 100 μg/mL, chloramphenicol 7.5 μg/mL (*L. monocytogenes*) or 20 μg/mL (*E. coli*). To generate an isogenic *hly* deletion mutant of *L. monocytogenes* LS741, the *hly* gene was deleted from the genome of *L. monocytogenes* LS741. The pKSV7 Δ*hly* plasmid was electroporated into LS741 and allelic exchange was performed as described previously^34,47^ to generate strain *L. monocytogenes* LS741 Δ*hly*.

### Cell culture

Bone marrow-derived macrophages (BMM) were cultured as previously described^48^. Briefly, 8–12 week old female BALB/c mice (The Jackson Laboratory) were euthanized and femurs removed. Bone marrow was then flushed from the femurs with Dulbecco’s Modified Eagle’s Medium (DMEM) (Mediatech) with 100 μg/mL penicillin-streptomycin. Cells were then cultured in BMM medium (DMEM supplemented with 10% fetal bovine serum (FBS; HyClone), 2 mM glutamine, 1 mM sodium pyruvate, 100 μg/mL penicillin-streptomycin, 55 μM β-mercaptoethanol, and 30% L-cell conditioned medium) in 150 mm non-tissue-culture treated petri dishes (Nalge Nunc International). On day 3, the culture medium was replaced with fresh BMM medium. On day 7, media was removed from the cells and BMM were harvested. Recovered BMM cells were then plated in antibiotic-free medium 18–24 hours prior to experiments as indicated or were frozen in FBS with 5% DMSO and aliquots stored in liquid nitrogen for use in the interferon (IFN)-γ ELISPOT assay. The human-derived epithelial cell line HeLa (ATCC CCL-2) was cultured in RPMI 1640 medium (Mediatech) supplemented with 10% FBS, 55 mM β-mercaptoethanol, 1 mM sodium pyruvate, 2 mM glutamine and 100 μg/mL penicillin-streptomycin. All cells were maintained at 37 °C in a 5% CO_2_-air atmosphere.

### Gentamicin protection assay

HeLa cells were seeded in 12-well tissue culture plates and grown to 80–90% confluency. On the day of infection, monolayers were washed twice with phosphate buffered saline (PBS) and bacteria from 16-hour cultures were added to the monolayers at a MOI of 50:1 in tissue culture media and incubated for 1 hour at 37 °C. After 1 hour of infection, the monolayers were washed three times with PBS and then extracellular bacteria were selectively killed by incubating infected monolayers for 1 hour in culture medium containing 30 μg/mL gentamicin. To quantify bacterial invasion of host cells, the monolayers were washed two times with PBS followed by lysis of monolayers with 1% Triton X-100 and plating of dilutions on agar plates for enumeration of viable intracellular bacteria.

### Plaquing assay

HeLa cells were seeded at 2 × 10^6^ cells per well in a 6-well plate and grown in RPMI 10% FBS for 18–24 hours. The indicated *L. monocytogenes* strains were grown for 16 hours in BHI at 30 °C. Two microliters of a 1:10 dilution of the bacterial culture was added to the HeLa cells in 2 mL of RPMI medium. The infected HeLa cells were incubated for 1 hour at 37 °C in a tissue culture incubator. Infected cells were then washed three times in PBS and overlaid with DMEM containing 5% FBS, 0.7% agarose and 30 μg/mL gentamicin. Cells were then incubated for 3 days to allow plaques to form and a second overlay of agarose and DMEM containing 400 μg/mL neutral red and 12 mM HCl was added. Twenty-four hours later, plaques were imaged and the relative plaque diameters were measured.

### *In vitro* growth in BHI broth and flagellar motility assays

Sixteen-hour cultures of the indicated strains were diluted 1:20 in duplicate in BHI and grown with shaking at 37 °C. At the indicated times, the optical density at 600 nm (OD_600_) of the bacterial cultures was measured or an aliquot of bacterial cultures was collected to enumerate bacterial colony forming units (CFU). To analyze the flagellar motility of *L. monocytogenes* strains, 10403S, LS741 and LS743 were spotted on a soft agar plate (1% tryptone, 0.25% NaCl, and 0.3% agar) and incubated at 30 °C for 16 hours.

### Intracellular growth assay

A total of 2 × 10^5^ HeLa cells were seeded into each well of a 24-well plate 18–24 hours prior to infection. The indicated *L. monocytogenes* strains were grown for 16 hours in 3 mL of BHI medium at 30 °C without shaking. The bacterial cultures were washed once with PBS and used for infection of host cells at a MOI of 10:1. At 1 hour post-infection, monolayers were washed three times with PBS and RPMI medium containing 30 μg/mL gentamicin was added to the infected cells. At the indicated time intervals, bacteria were collected by lysing host cells in 1 mL of PBS containing 1% Triton X-100. Dilutions of the lysates were then plated on LB agar plates and incubated 24–36 hours at 37 °C to enumerate bacterial colony forming units (CFU).

### *In vivo* virulence studies and LD_50_ determination

For animal infections with *L. monocytogenes*, 6–8 week old female BALB/c mice were purchased from The Jackson Laboratory. Eight to ten week old BALB/c mice were injected intravenously with 1–2 × 10^4^ bacteria/mouse of 10403S, LS741, LS743, or the LS741 Δ*hly* strain. In some experiments, mice were infected by orogastric inoculation with 3–4 × 10^9^ bacteria of the indicated strains. At 72 hours post-infection, mice were euthanized by exposure to CO_2_ followed by cervical dislocation and organs were collected for bacterial CFU enumeration by plating dilutions of organ homogenates.

In separate experiments, BALB/c mice were infected intravenously with 2 × 10^3^, 4 × 10^3^, 6 × 10^3^, 8 × 10^3^, or 1 × 10^4^ CFU of *L. monocytogenes* LS741 bacteria per mouse. Each group included 4 mice and experiments were repeated twice. The CFU of injected bacteria were confirmed by plating serial dilutions of the inoculums on BHI agar. Mice were monitored twice daily for signs of disease and moribund animals were euthanized on day 3 post-infection. Surviving mice were monitored twice daily for an additional seven days for signs of disease and moribund animals were euthanized. Ten days after infection, the 50% lethal dose (LD_50_) was estimated on the basis of mouse survival data^31^.

All animal care and experiments were conducted at the Harvard Institutes of Medicine BL-2 Animal Facility. All animal care and experiments were conducted in compliance with the Association for Assessment and Accreditation of Laboratory Animal Care regulations. All experimental protocols were approved by the Harvard Medical School Institutional Animal Care and Use Committee and were in compliance with all federal, state and local laws.

### Mice immunization and challenge

Eight-week-old female BALB/c mice were immunized intravenously with 0.1 LD_50_ of the indicated *L. monocytogenes* strains in 200 μL of PBS (pH 7.2). The CFU of the infection inoculums were confirmed by plating serial dilutions on BHI agar. Mice were monitored daily for signs of disease after immunization. Twenty-eight days later, mice were challenged with 1 × 10^5^ CFU of the indicated strains in 200 μL of PBS (pH 7.2) via tail vein injection. Mice were monitored twice daily for signs of disease. At 48 hours post-challenge, mice were euthanized and bacterial burdens in the liver, spleen, and brain were enumerated by plating dilutions of organ homogenates on BHI agar.

### Determination of hemolytic activity

*L. monocytogenes* strains were grown in BHI for 16 hours at 30 °C without shaking. Cultures were then diluted 1:10 into fresh LB medium and grown with vigorous shaking for 5 hours at 37 °C. After bacterial growth, 1 ml aliquots of bacteria were centrifuged at 13,000 rpm in a microcentrifuge for 2 min and the supernatants were saved for hemolytic activity determination. Hemolytic activity in culture supernatants was determined as previously described^32,49^ with some modifications and is expressed as the reciprocal of the supernatant dilution required to lyse 50% of sheep erythrocytes. Briefly, two-fold serial dilutions of culture supernatants were made in PBS (pH 5.5) in microcentrifuge tubes. Aliquots of 900 μL of each dilution were incubated for 30 minutes at 37 °C. Subsequently, 100 μL of a 5% solution of sheep red blood cells (Hemostat Laboratories) was added to each sample. After an additional 30-minute incubation at 37 °C, the tubes were subjected to centrifugation at 13,000 rpm for 1 minute. A 100 μL volume of the supernatant was transferred to a 96-well flat-bottom microtiter plate (Nalge Nunc International) and the absorbance at 541 nm of each well was measured in a VERSAmax microplate reader with SoftMax Pro v1.2 software (Molecular Devices). Numbers of hemolytic units (HU) were defined as the reciprocal of the dilution of culture supernatant that yielded 50% lysis of sheep red blood cells. To determine 50% lysis, 100 μL of 10% Triton X-100 was added to 850 μL of PBS (pH 5.5) to which 50 μL of a 5% solution of sheep red blood cells was previously added and incubated for 30 minutes at 37 °C. The assay was repeated 5 times with duplicates. The results presented represent the mean ± SD.

### Isolation of *Listeria*-specific CD8^+^ T cells

*Listeria*-specific T cells were isolated from mouse spleens 7 days after tail vein injection of BALB/c mice with 0.1 LD_50_ of the *L. monocytogenes* strain. CD8^+^ T cells were enriched from pooled single cell suspensions of spleens isolated from immunized mice by magnetic separation using the Dynabeads Untouched™ Mouse CD8 Cells Kit (Thermo Fisher Scientific) according to the manufacturer’s protocol. Briefly, isolated spleens were dissociated by compression using a 70 μm cell strainer. Dissociated splenocytes were placed in a 50 mL conical tube and centrifuged at 250 × *g* for 10 minutes. Pelleted cells were resuspended in 8 mL of ACK lysis buffer (Life Technologies) and incubated for 3 minutes at 37 °C. Twenty milliliters of PBS was added and the splenocytes were re-pelleted by centrifuging at 250 × *g* for 10 minutes. Pelleted splenocytes were resuspended in 500 μL of isolation buffer (DPBS, supplemented with 2 mM EDTA and 1% BSA), 100 μL of heat-inactivated FBS and 100 μL of Antibody-Mix were added and the splenocytes were then incubated for 20 minutes at 4 °C. Cells were washed by adding 10 mL of isolation buffer and cells centrifuged at 350 × *g* for 8 minutes at 2–4 °C. Pelleted cells were resuspended in 5 mL of isolation buffer and mixed with 1 mL of isolation buffer-washed Mouse Depletion Dynabeads. The suspension was incubated for 15 minutes at room temperature (18–25 °C) with gentle tilting and rotation using a nutator. The suspension was transferred to a 15 mL conical tube and the tube was placed within a MPC-6 magnetic particle concentrator (Dynal) for 2 minutes. The supernatant was then transferred to a new 15 mL conical tube and the CD8^+^ T cells were isolated by centrifugation at 250 × *g* for 10 min. CD8^+^ T cells were resuspended in complete RPMI (RPMI 1640 medium supplemented with 10% FBS, 55 mM β-mercaptoethanol, 1 mM sodium pyruvate and 2 mM glutamine) with 100 μg/mL penicillin-streptomycin.

### IFN-γ ELISPOT assay

The IFN-γ enzyme linked immunospot (ELISPOT) assays were performed using the Mouse IFN-γ ELISPOT Ready-SET-Go! kit according to the manufacturer’s instructions (Thermo Fisher Scientific). Briefly, 96-well MultiScreen Filter Plates (Merck Millipore) were pre-activated with 70% alcohol for 2 minutes and then washed 3 times with dH_2_O. ELISPOT plates were coated with 100 μL of 1 μg/ml anti-mouse IFN-γ mAb for 16 hours at 4 °C. The next day, plates were washed with PBST (PBS with 0.05% Tween-20) and then blocked with 200 μL of RPMI media with 10% FBS for 1 hour at room temperature (18–25 °C) before use. Thawed BMM (1 × 10^5^ cells/well) were added to the ELISPOT plates in 50 μL of complete RPMI with no antibiotics. *L. monocytogenes* was then immediately added at a MOI of 5:1 (5 × 10^5^ bacteria/well) in 50 μL of PBS and incubated with the BMM for 1 hour at 37 °C in a 5% CO_2_-air atmosphere. Fifty microliters of complete RPMI containing gentamicin (50 μg/ml) was added to the BMM wells infected with bacteria and the ELISPOT plates were incubated for an additional 3 hours at 37 °C in a 5% CO_2_-air atmosphere. Separately, 50 μL of LLO 91–99 or p60 217–225 peptides (100 nM) in complete RPMI media with no antibiotics were added to triplicate wells of BMM uninfected with bacteria and incubated for 2 hours at 37 °C in a 5% CO_2_-air atmosphere. Fifty microliters of *Listeria*-specific CD8^**+**^ T cells (1 × 10^5^ cells/well) were then added to the bacteria or peptide-pulsed BMM and the ELISPOT plates were then incubated 17–20 hours. The next day, the ELISPOT plates were rinsed with PBST and 100 μL of PBST with 5% FBS containing 1 μg/ml biotinylated anti-mouse IFN-γ mAb was added and the plates incubated for 2 hours at room temperature (18–25 °C). The ELISPOT plates were then washed 4X with 200 μL/well of PBST followed by addition of 100 μL/well of the ELISPOT kit strepavidin-HRP solution (Thermo Fisher Scientific). The plates were then incubated for 45 minutes at room temperature (18–25 °C). The ELISPOT plates were then washed 4X with 200 μL/well of PBST and 2X with 200 μL/well of PBS. After washing, the AEC Staining Kit was used to develop the immunospots (Invitrogen). Finally, spots were imaged and enumerated using an ImmunoSpot Analyzer with ImmunoSpot v5.1.36 software (Cellular Technology Limited). The LLO 91–99 (GYKDGNEYI) and p60 217–225 (KYGVSVQDI) peptides were synthesized with >98% purity (Research Genetics).

### Statistical analysis

Statistical analysis for gentamicin protection and IFN-γ ELISPOT assays was performed using the Student’s *t-*test (two-tailed, unpaired). Statistical analysis for *in vivo* virulence studies was performed using the Mann-Whitney U-test. Differences were considered significant at *P* < 0.05.

## Supplementary information

Supplementary Information
