# Supplementary material for: Characterization of the pathogenesis and immune response to Listeria monocytogenes strains isolated from a sustained national outbreak
Source: Sci Rep. 2019 Dec 20;9:19587. doi: 10.1038/s41598-019-56028-3 (PMC6925182; doi:10.1038/s41598-019-56028-3)
Supplement: Supplementary file 1 — Supplementary Information [file 41598_2019_56028_MOESM1_ESM.pdf]

Supplementary Information to

Characterization of the pathogenesis and immune response to *Listeria monocytogenes* strains isolated from a sustained national outbreak

Pallab Ghosh, Yan Zhou, Quentin Richardson and Darren E. Higgins

Supplementary Figures

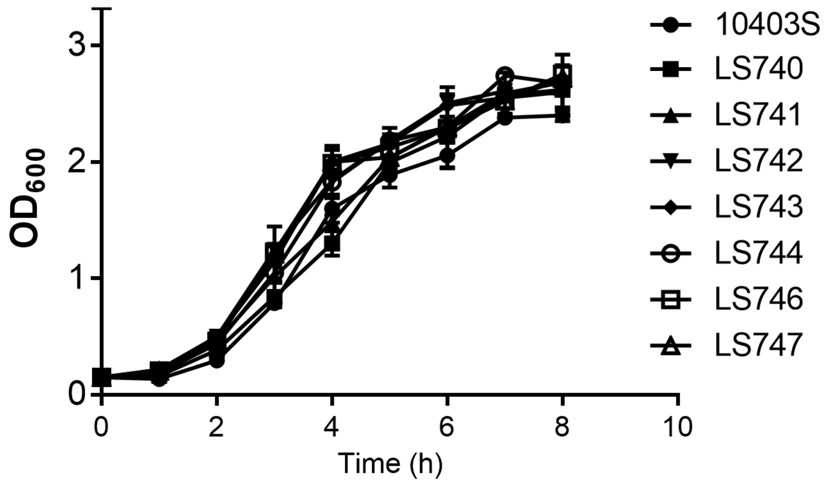

Supplementary Figure S1. *In vitro* growth of *L. monocytogenes* strains in BHI broth. Bacterial cultures were grown shaking at 37°C for 8 hours and the OD_{600} was measured at 60-minute intervals. Data represents the mean ±SD of three experiments.

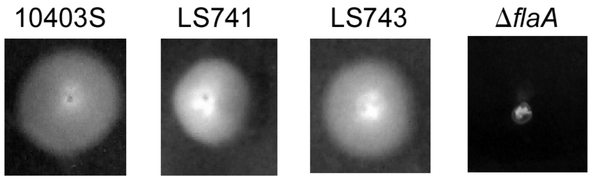

Supplementary Figure S2. Motility of *L. monocytogenes* strains. Flagellar motility of *L. monocytogenes* 10403S, LS741, LS743, and the flagellar motility-deficient EGDe Δ*flaA* (Δ*flaA*) strains are shown on 0.3% agar medium grown at 30°C for 16 hours. Images shown are representative of two separate experiments with similar results.

```
10403S PrfA      1   MNAQAEEFKKYLETNGIKPKQFHKKELIFNQWDPQEYCIFLYDGITKLTSISENGTIMNL  60
LS741 PrfA       1   MNAQAEEFKKYLETNGIKPKQFHKKELIFNQWDPQEYCIFLYDGITKLTSISENGTIMNL  60
LS743 PrfA       1   MNAQAEEFKKYLETNGIKPKQFHKKELIFNQWDPQEYCIFLYDGITKLTSISENGTIMNL  60
Seq. identities      MNAQAEEFKKYLETNGIKPKQFHKKELIFNQWDPQEYCIFLYDGITKLTSISENGTIMNL

10403S PrfA     61   QYYKGAFVIMSGFIDTETSVGYYNLEVISEQATAYVIKINELKELLSKNLTHFFYVFQTL  120
LS741 PrfA      61   QYYKGAFVIMSGFIDTETSVGYYNLEVISEQATAYVIKINELKELLSKNLTHFFYVFQTL  120
LS743 PrfA      61   QYYKGAFVIMSGFIDTETSVGYYNLEVISEQATAYVIKINELKELLSKNLTHFFYVFQTL  120
Seq. identities      QYYKGAFVIMSGFIDTETSVGYYNLEVISEQATAYVIKINELKELLSKNLTHFFYVFQTL

10403S PrfA    121   QKQVSYSLAKFNDFSINGKLGSICGQLLILTYVYGKETPDGIKITLDNLTMQELGYSSGI  180
LS741 PrfA     121   QKQVSYSLAKFNDFSINGKLGSICGQLLILTYVYGKETPDGIKITLDNLTMQELGYSSGI  180
LS743 PrfA     121   QKQVSYSLAKFNDFSINGKLGSICGQLLILTYVYGKETPDGIKITLDNLTMQELGYSSGI  180
Seq. identities      QKQVSYSLAKFNDFSINGKLGSICGQLLILTYVYGKETPDGIKITLDNLTMQELGYSSGI

10403S PrfA    181   AHSSAVSRIISKLKQEKVIVYKNSCFYVQNLDYLKRYAPKLDEWFYLACPATWGKLN     237
LS741 PrfA     181   AHSSAVSRIISKLKQEKVIVYKNSCFYVQNLDYLKRYAPKLDEWFYLACPATWGKLN     237
LS743 PrfA     181   AHSSAVSRIISKLKQEKVIVYKNSCFYVQNLDYLKRYAPKLDEWFYLACPATWGKLN     237
Seq. identities      AHSSAVSRIISKLKQEKVIVYKNSCFYVQNLDYLKRYAPKLDEWFYLACPATWGKLN
```

Supplementary Figure S3. Protein sequence alignment of PrfA in *L. monocytogenes* strains 10403S, LS741 and LS743. The known PrfA* mutations[1,2] do not appear in 10403S or the LS741 and LS743 outbreak strains.

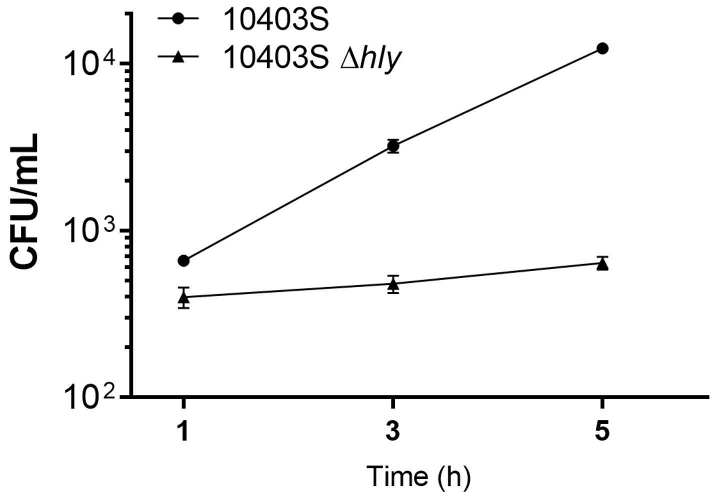

Supplementary Figure S4. Intracellular growth of *L. monocytogenes* in HeLa cells. HeLa cells were infected with the indicated strains for 1 hour. Gentamicin was then added and at 2-hour intervals post-infection, HeLa cells were lysed and intracellular bacteria were enumerated by plating dilutions of lysates. Data presented is the mean ±SD of three independent experiments performed in duplicate.

Supplementary Table S1. Characteristics of the *L. monocytogenes* strains used in this study.

Strain or isolate	Alternative name	PFGE pattern#	ST**	Serotype	Outbreak associated	Source	Reference(s)	Biosample or accession no.
LS740	CFSAN012827	II	NA	1/2b	Yes, cantaloupe outbreak in 2011	Human	[3]	SAMN02769788
LS741	CFSAN012828	II	5	1/2b	Yes, cantaloupe outbreak in 2011	Human	[3]	SAMN02769789, AAALMK000000000.1
LS742	CFSAN012829	I	NA	1/2a	Yes, cantaloupe outbreak in 2011	Human	[3]	SAMN02769790
LS743	CFSAN012830	I	29	1/2a	Yes, cantaloupe outbreak in 2011	Human	[3,4]	SAMN02769791, JRZB00000000.1
LS744	CFSAN012831	IV	NA	1/2a	Yes, cantaloupe outbreak in 2011	Human	[3]	SAMN02769792
LS746	CFSAN012832	III	NA	1/2a	Yes, cantaloupe outbreak in 2011	Human	[3]	SAMN02769793
LS747	CFSAN012833	IV	NA	1/2a	Yes, cantaloupe outbreak in 2011	Human	[3]	SAMN02769794
10403S	N/A	N/A	85	1/2a	No	Human, lab reference strain	[5,6]	SAMN02641382, NC_017544
10403S Δ*hly*	DP-L2161	N/A	85	1/2a	N/A		[7]	N/A
EGDe Δ*flaA*	DH-L1042	N/A	35	1/2a	N/A	Lab reference strain	[8]	N/A
LS741 Δ*hly*	DH-L2148	II	5	1/2b	N/A		This study	N/A

#Pulsed-field gel electrophoresis (PFGE) pattern combinations based on the restriction enzymes *AscI* and *ApaI* are reported as described[3].

**ST, sequence type based on a 7-gene multilocus sequence typing (MLST) comparison as observed in the Institut Pasteur *L. monocytogenes* MLST database (https://bigsdb.pasteur.fr/listeria/listeria.html).

NA: not analyzed

N/A: not applicable

Supplementary Table S2. *Escherichia coli* strains and plasmids used in this study.

Strain or plasmid	Strain genotype or plasmid description	Reference(s)
pKSV7	Allelic exchange vector for *L. monocytogenes*	9
pKSV7 Δ*hly*	Allelic exchange vector for the in-frame deletion of *hly* in LS741	7
DP-E2154	*E. coli* harboring pKSV7 Δ*hly*	7

Supplementary References

1 Eiting, M., Hageluken, G., Schubert, W. D. & Heinz, D. W. The mutation G145S in PrfA, a key virulence regulator of *Listeria monocytogenes*, increases DNA-binding affinity by stabilizing the HTH motif. *Mol. Microbiol.* **56**, 433-446 (2005).

2 Becavin, C. *et al.* Comparison of widely used *Listeria monocytogenes* strains EGD, 10403S, and EGD-e highlights genomic variations underlying differences in pathogenicity. *mBio* **5**, 10.1128/mBio.00969-00914 (2014).

3 Laksanalamai, P. *et al.* Genomic characterization of *Listeria monocytogenes* strains involved in a multistate listeriosis outbreak associated with cantaloupe in US. *PLoS One* **7**, e42448; 42410.41371/journal.pone.0042448 (2012).

4 Burall, L. S., Grim, C., Gopinath, G., Laksanalamai, P. & Datta, A. R. Whole-genome sequencing identifies an atypical *Listeria monocytogenes* strain isolated from pet foods. *Genome Announc.* **2**, e01243-01214; 01210.01128/genomeA.01243-01214 (2014).

5 Edman, D. C., Pollock, M. B. & Hall, E. R. *Listeria monocytogenes* L forms. I. Induction maintenance, and biological characteristics. *J. Bacteriol.* **96**, 352-357 (1968).

6 Bishop, D. K. & Hinrichs, D. J. Adoptive transfer of immunity to *Listeria monocytogenes*. The influence of *in vitro* stimulation on lymphocyte subset requirements. *J. Immunol.* **139**, 2005-2009 (1987).

7 Jones, S. & Portnoy, D. A. Characterization of *Listeria monocytogenes* pathogenesis in a strain expressing perfringolysin O in place of listeriolysin O. *Infect. Immun.* **62**, 5608-5613 (1994).

8 Gründling, A., Burrack, L. S., Bouwer, H. G. & Higgins, D. E. *Listeria monocytogenes* regulates flagellar motility gene expression through MogR, a transcriptional repressor required for virulence. *Proc. Natl. Acad. Sci. U.S.A.* **101**, 12318-12323 (2004).

9 Smith, K. & Youngman, P. Use of a new integrational vector to investigate compartment-specific expression of the *Bacillus subtilis* spoIIM gene. *Biochimie* **74**, 705-711 (1992).
